# The Impact of Health Status, Chronic Conditions, and Mental Fatigue on College Students’ Grade Expectations in Online Courses

**DOI:** 10.3390/ejihpe15070118

**Published:** 2025-06-25

**Authors:** Fethi Ahmet Inan, Deniz Unal, Fatemeh Marzban, Edwin Teye Sosi, Gail Alleyne Bayne

**Affiliations:** Educational Instructional Technology, College of Education, Texas Tech University, Lubbock, TX 79409, USA; dnal@ttu.edu (D.U.); fatemeh.marzban@ttu.edu (F.M.); esosi@ttu.edu (E.T.S.); gail.alleyne-bayne@ttu.edu (G.A.B.)

**Keywords:** mental fatigue, chronic conditions, perceived health, student achievement

## Abstract

This study explores the impact of mental fatigue, health status, and chronic conditions on college students’ grade expectations in online courses. Data were gathered from 418 undergraduate students through an online survey assessing mental fatigue and other relevant variables. Path analysis was used to examine the relationships between these variables and the proposed research model. Key findings revealed that mental fatigue had a significant negative direct effect on expected grades, indicating that higher mental fatigue was associated with lower grade expectations. Health status demonstrated a positive indirect effect on grade expectations by negatively impacting mental fatigue. Chronic conditions had a significant negative direct impact on expected grades and a significant negative direct impact on health status, but no direct significant impact on mental fatigue. These results emphasize the critical need for comprehensive support services that address mental fatigue and health conditions to enhance student well-being and academic progress and performance in online learning environments. Additionally, the findings suggest the need for inclusive course designs that minimize cognitive overload and provide flexible support for students with chronic conditions.

## 1. Introduction

### 1.1. Online Learning

Online education has been experiencing significant growth in the 21st century, and this trend will likely continue (Xu & Xu, 2019). The National Center for Education Statistics reported that the percentage of undergraduate students enrolled in at least one online education course was 61% (9.4 million students) in 2021 (National Center for Education Statistics, 2023). Due to the ongoing demand for flexible online learning options, many institutions are expanding their online course offerings (Garrett et al., 2023). Today, most institutions are choosing to take a more proactive approach to marketing their online programs as part of their institutional strategy. In addition, there are ongoing changes in delivery formats and methods, as well as adaptive and flexible online course and degree offerings to meet the diverse needs of the student market (Bashir et al., 2021).

The COVID-19 pandemic spurred a rapid global shift to online learning, dramatically increasing demand for online courses (Reuge et al., 2021). This abrupt transition highlighted existing challenges in online learning and significantly impacted unprepared teachers and students required to take online courses. This sudden change posed substantial difficulties for students, potentially harming their physical, emotional, and mental well-being (Mosleh et al., 2022). Research during the pandemic revealed various mental health challenges among online learners, including depression, stress, anxiety, boredom, and disengagement (Odriozola-González et al., 2020; Planchuelo-Gómez et al., 2020; Son et al., 2020).

The inherent challenges of online learning and its evolving nature require a deeper understanding of student well-being issues. These issues significantly influence their academic progress and performance. For example, students who suffer from mental health issues that impact their learning can lead to poorer academic performance (Richardson, 2015). Similarly, chronic conditions (such as autoimmune diseases, cancer, diabetes, etc.) and/or poor health may also have an impact on students’ academic learning outcomes (Shrout & Weigel, 2022). However, it appears that factors related to a student’s well-being, such as the proportion and prevalence of online students with various physical and mental health issues, are not commonly recognized or considered significant for students who engage in online learning (Chung & McKenzie, 2020; Van Cutsem et al., 2017).

### 1.2. Student Health and Well-Being in Online Learning

Student health and well-being play a pivotal role in shaping academic expectations and outcomes, especially in the demanding environment of online learning (C. E. Basch, 2011). A holistic view of health including physical, mental, and health conditions, all of which can significantly impact cognitive functioning and academic performance (Norozi, 2023). When students experience health challenges, it can manifest in various ways, including increased mental fatigue and the exacerbation of chronic conditions. Research suggests that physically and mentally healthier students tend to have higher academic expectations and learning outcomes (C. E. Basch, 2011). This relationship is supported by studies highlighting the direct and indirect link between health, academic expectations, and performance in various educational settings (Bradley & Greene, 2013).

#### 1.2.1. Health Status

Research indicates a strong association between poor health status and increased mental fatigue, leading to impaired cognitive processing and learning outcomes (Zdun-Ryżewska et al., 2021). Additionally, somatic disorders and psychological distress have been linked to increased mental fatigue (Mosleh et al., 2022). Mental conditions like anxiety and depression, as well as prolonged stress, can also contribute to cognitive resource depletion and fatigue (Jackson, 2014; Zdun-Ryżewska et al., 2021). Poor health status can lead to mental fatigue, which can mediate the relationship between health status and academic outcomes by affecting cognitive processes and learning (Zdun-Ryżewska et al., 2021). Furthermore, mental fatigue arising from coping challenges with health conditions can lower academic expectations (Milyavskaya et al., 2021). Consequently, poorer health can indirectly impact expected grades by increasing mental fatigue and diminishing academic expectations.

#### 1.2.2. Mental Fatigue

In online learning, mental fatigue and well-being are crucial factors impacting a student’s participation, performance, learning, and retention. Mental fatigue can function as an underlying construct, mediating the relationship between these factors and learning outcomes (Earl et al., 2023). Mental fatigue is characterized as a state of exhaustion mainly caused by prolonged mental activities and sustained attention on cognitive tasks (National Library of Medicine, 2021). The depletion of cognitive resources over time is considered a primary source of mental fatigue (Balkin & Wesensten, 2011). Its distinct nature from physical fatigue lies in the significant burden it places on cognitive and neural systems (Van Cutsem et al., 2017). Various direct and indirect factors contribute to mental fatigue, influenced by an individual’s overall health and well-being (Lister et al., 2023).

Mental fatigue presents significant cognitive and behavioral challenges, adversely impacting performance in complex cognitive tasks (Borragán et al., 2017; Van Cutsem et al., 2017). Individuals experiencing mental fatigue often exhibit impaired attention and executive functions, affecting their ability to focus on relevant information and suppress irrelevant information. This leads to heightened distraction, diminished cognitive control, and reduced information processing (Sy et al., 2021). Consequently, reduced performance, slower reaction times, and a general sense of weariness are frequently observed in individuals with mental fatigue, affecting various aspects of cognitive functioning such as concentration, attention, and decision-making. As a result, mental fatigue may lead to poorer learning outcomes, including lower grades and challenges in completing courses (Richardson, 2015).

#### 1.2.3. Chronic Conditions

Living with a chronic condition presents numerous challenges that can significantly affect a student’s overall health status. Research has shown that chronic conditions, such as autoimmune diseases, cancer, and mental health conditions, can negatively impact both physical and mental health (Shrout & Weigel, 2022). Chronic conditions are also frequently associated with increased levels of both physical and mental fatigue (Vaes et al., 2022). Studies have found that students with a higher number of chronic conditions and more severe symptoms often experience heightened mental fatigue, which can impair their cognitive abilities (Shrout & Weigel, 2022).

Studies suggest that chronic conditions can significantly impact students’ academic expectations (Cerqueira et al., 2022). According to the American College Health Association, 40% of college students with persistent or chronic conditions reported that their health conditions had a negative impact on their academic performance (American College Health Association, 2023). Students with chronic conditions often have lower grade expectations and are more likely to experience academic difficulties, including lower grades, increased absenteeism, and a higher likelihood of dropping out of school (Shrout & Weigel, 2022; Wisk & Weitzman, 2017). This negative impact on expected grades can be attributed to various factors, including the challenges of managing the condition alongside academic responsibilities, lower expectations from parents and teachers, and the cumulative effects of missed classes and assignments (Cerqueira et al., 2022; Wisk & Weitzman, 2017).

### 1.3. Path Model

**Figure 1:**
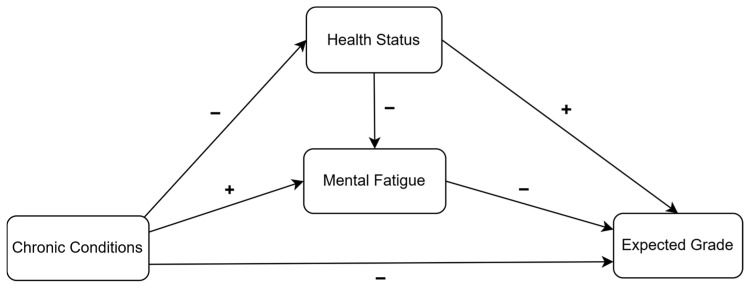
Proposed path model.

The purpose of this study is to explore how health, chronic conditions, and mental fatigue influence online students’ grade expectations, using path analysis to explain these relationships. The proposed model builds on existing research and addresses the gap in understanding mental fatigue in online learning contexts. It includes both exogenous variables (chronic condition) and endogenous variables (health status, mental fatigue, and expected grade), as depicted in Figure 1. In the model, a positive impact is indicated when one variable increases and the value of other variable also increases (e.g., they move in the same direction). Conversely, a negative impact is indicated when one variable increases and the other variable decreases (e.g., they move in opposite directions). These terms do not indicate whether the impact is good or bad; they simply describe the direction of the relationship.

In the model, it was hypothesized that chronic conditions have a negative impact on mental fatigue (Vaes et al., 2022), perceived health status (Joe et al., 2009), and anticipated grade (Koivusilta et al., 2022). Students’ mental fatigue was inversely correlated with perceived health status, suggesting that students who feel healthier are likely to experience less mental fatigue (Armand et al., 2021). According to the model, expected grade was hypothesized to be directly influenced by mental fatigue and the relationship is negative, suggesting that higher mental fatigue leads to lower expected grade expectations (Li et al., 2022). The model also hypothesized that chronic conditions and health status indirectly influence expected grade, as these influences are mediated by mental fatigue (Kirkpatrick, 2020; Milyavskaya et al., 2021).

Hypothesized direct and indirect impacts guiding the study include:

**H1.**  *Mental fatigue directly negatively impacts expected grade.*

**H2a.**  *Health status directly negatively impacts mental fatigue.*

**H2b.**  *Health status directly positively impacts expected grade.*

**H2c.**  *Health status indirectly impacts expected grade through mental fatigue.*

**H3a.**  *Chronic conditions directly negatively impact health status.*

**H3b.**  *Chronic conditions directly positively impact mental fatigue.*

**H3c.**  *Chronic conditions directly negatively impact expected grade.*

**H3d.**  *Chronic conditions indirectly impact mental fatigue and expected grade.*

## 2. Materials and Methods

### 2.1. Participants

The study included 418 undergraduate students who had taken an online course and had completed data on the variables studied. Participants, recruited via the Qualtrics Sample Panel, were required to be at least 18 years old and enrolled in a fully online course (defined as all activity being online with no required face-to-face or on-campus sessions) at a 4-year US university. Qualtrics partners randomly selected potential participants from their panel, proportionally representing the general population and then randomized further before survey distribution. Participants were enrolled in a wide range of online courses across various higher education institutions in the United States, studying diverse subjects.

The majority were female (83.0%) aged 18–50 years (M = 26.96, SD = 7.49). Participants self-identified with the following ethnicities: White (66.0%), Black/African American (15.6%), Hispanic/Latino (10.5%), Asian (3.1%), Multiple (2.2%), Indian/Alaskan Native American (1.7%), and Other (1.0%). Most students are in their first year (26.6%) or second year (40.4%) of college. Regarding employment status, a substantial proportion of participants were employed full-time (32.8%) or part-time (30.9%). With regard to family responsibilities, about half of the participants (47.6%) reported that they had a dependent at home who was under 18 years old.

### 2.2. Instruments

Demographics and Background Questionnaire. The questionnaire was used to collect information about students’ demographic characteristics, academic background, and health status. Specifically, the questionnaire included items about students’ self-assessment of their general health using a rating scale from poor to excellent, whether they had a doctor-diagnosed chronic medical condition, and their expected grade letter in the online course.

Student Mental Fatigue Survey (SMFS). The SMFS was used to obtain information on students’ perceptions of mental fatigue while taking online courses. The survey had 8 items on a 5-point Likert scale (1 = strongly disagree to 5 = strongly agree), with an average score for the entire scale. In a published validation study, the survey’s reliability was reported to be high (Cronbach alpha = 0.91) (Bayne & Inan, 2022). A confirmatory factor analysis (CFA) supported the hypothesized single-factor structure of the 8-item survey. Key fit indices suggested an acceptable to good model fit (CFI = 0.966; TLI = 0.953; RMSEA = 0.089; SRMR = 0.031). Standardized factor loadings were substantial, ranging from approximately 0.59 to 0.85. The scale exhibited high internal consistency (McDonald’s ω = 0.915).

### 2.3. Procedure

Institutional review board approval was obtained from Human Research Protection Program of Texas Tech University (IRB2018-872). All procedures performed in this study were in accordance with the ethical standards of the institutional and national research committee and with the 1964 Helsinki declaration and its later amendments or comparable ethical standards. All participants provided informed consent prior to participation. Panel sample participants were recruited for the study by Qualtrics Panels, LLC. Participants were given an online survey and asked to complete a series of questions, including their self-assessment of their level of mental fatigue while participating in an online course.

### 2.4. Data Analysis

Path analysis was used to explain the relationship by analyzing the study data within the variables of the proposed model. Path analysis allows researchers to conceptualize and estimate complex relationships between observed variables (Barbeau et al., 2019). This technique helps to compare the magnitude of the relationships between variables and has implications for proposed hypotheses in research. Path analysis can decompose the correlation between the variables into direct and indirect effects of the independent variables on student performance (Kline, 2016). This approach helps in the investigation of complex interrelations that unveil essential information and associations between independent and dependent variables that may be overlooked within the framework of traditional multiple regressions (Barbeau et al., 2019; Kline, 2016). STATA 18 was used for descriptive statistics and Mplus 8.11 for estimation of path model parameters.

## 3. Results

### 3.1. Descriptive Results

An examination of the descriptive analysis results indicated that students had high overall grade expectations (M = 4.21), suggesting that most students were aiming for an A or B letter grade. In terms of mental fatigue (M = 3.21), the scores indicated that students were slightly fatigued. Regarding health status (M = 3.25), students reported being in good health on average. With regard to chronic conditions, a small portion of participants (17.7%) had a diagnosis. Table 1 below presents a descriptive overview of the variables involved in the path model. Correlations between each variable are shown at the intersection of their respective rows and columns. Additionally, means and standard deviations are provided for each variable (see Table 1).

### 3.2. Path Model Estimates

In the path analysis, the factors examined included expected grade, mental fatigue, and health status as endogenous variables, while chronic conditions were an exogenous variable. In addition, mental fatigue and health status served as mediator variables in the model. The size and direction of effects were determined from standardized path coefficients (betas) obtained using Mplus Version 8.11 (Muthén & Muthén, 2017). Indirect effects were estimated, and their statistical significance was assessed by calculating standard errors and *p*-values using the delta method (MacKinnon, 2008), as implemented in Mplus. Statistical significance for all direct and indirect effects was evaluated using *p*-values, with effects considered significant at alpha levels of 0.05 and 0.01. The specified path model was saturated, and consequently demonstrated an excellent fit to the data: CFI = 1.00; TLI = 1.00; RMSEA = 0.00; and SRMR = 0.00 (Kline, 2016). Furthermore, the AIC and BIC values were 3111.65 and 3160.08, respectively.

The hypothesized factors in the model explained approximately 11% of the variance in students’ expected grades. Consistent with the conceptual model, mental fatigue showed a significant negative direct effect (beta = −0.27) on expected grades, which is considered a strong effect. Health status, on the other hand, had a positive total effect (beta = 0.15) on expected grades. Chronic conditions had a significant negative direct effect (beta = −0.11) and a significant negative total effect (beta = −0.14) on students’ expected grades. The variables in the model also explained 5% of the variance in mental fatigue. As hypothesized, health status (beta = −0.23) had a significant negative direct effect on mental fatigue. While chronic conditions did not directly influence mental fatigue, they had a significant negative effect (beta = −0.23) on health status, accounting for 5% of its variance. The path analysis revealed several noteworthy indirect (mediation) effects. The results highlighted a substantial indirect effect from health status to expected grades (beta = 0.06). In evaluating the pathways from chronic conditions to expected grades, however, the total indirect effect was not significant. The analysis also demonstrated a significant indirect effect (beta = 0.05) from chronic conditions to mental fatigue, operating through health status. The standardized regression coefficients (beta) and the coefficients of determination (*R*^2^) for endogenous variables are presented in Table 2.

**Figure 2:**
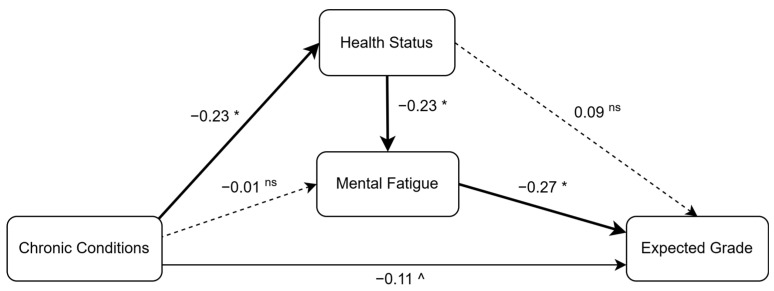
Path model estimates. Note: * *p* < 0.01, ^ *p* < 0.05, ^ns^ = Not Significant.

The finding sheds light on the complex interplay between chronic conditions, health, mental fatigue, and a student’s expected grade in online courses (see Figure 2). Here is a breakdown of the key findings along with the supported and unsupported hypothesis from the model estimation:

Supported

**H1.**  *Mental fatigue directly negatively impacts expected grade.*

**H2a.**  *Health status directly negatively impacts mental fatigue.*

**H2c.**  *Health status indirectly impacts expected grade through mental fatigue.*

**H3a.**  *Chronic conditions directly negatively impact health status.*

**H3c.**  *Chronic conditions directly negatively impact expected grade.*

Partially Supported

**H3d.**  *Chronic conditions indirectly impact mental fatigue and expected grade.*

Not Supported

**H2b.**  *Health status directly positively impacts expected grade.*

**H3b.**  *Chronic conditions directly positively impact mental fatigue.*

## 4. Discussion

This study investigates the impact of student-level factors, such as mental fatigue, health status, and chronic conditions, on students’ grade expectations. Among these factors, mental fatigue stands out as a significant determinant, negatively influencing expected grades. This indicates that students with higher mental fatigue tend to have lower expectations for their academic performance. These findings align with previous research that higher levels of mental fatigue are frequently associated with poorer learning and academic outcomes (Earl et al., 2023; Sievertsen et al., 2016). When students experience mental fatigue or mental tiredness, this impairs learners’ ability to focus on crucial elements of targeted tasks and their decision-making abilities (Sievertsen et al., 2016). As a result, their efficiency and work quality may decline, leading to difficulties in meeting deadlines and completing assignments (Febiyani et al., 2021). The impact of mental fatigue on grade expectation can be explained by cognitive load theory (Sweller, 1988). This theory posits that a student has limited cognitive resources. In online learning environments, where students are expected to manage multiple tasks simultaneously, increased mental fatigue may deplete these resources, leading to impaired cognitive processing and reduced grade expectation.

The findings indicated that health status does not directly affect academic outcome expectations, suggesting the involvement of other mediating factors. Indirectly, health status positively influenced expected grades by decreasing mental fatigue, which had a significant negative impact on expected grades. These results align with previous studies suggesting that better student health leads to lower levels of mental fatigue (Finsterer & Mahjoub, 2014). Research shows that even minor deterioration in health or health-related quality of life, accompanied by complaints of fatigue, has a significant impact on daily functioning, school participation, and meeting academic requirements (Zdun-Ryżewska et al., 2021). These findings support research on stress and coping, indicating that prolonged mental strain due to health status can influence individuals’ mental resources. Specifically, the transactional model of stress and coping (Lazarus & Folkman, 1984) posits that individuals perceive and respond to stressors in their environment. In this context, coping with poor health can lead to mental fatigue, as individuals expend mental energy managing their health issues. The findings also align well with Self-Regulation Theory (Bandura, 1991), which suggests that students with better health are more capable of self-regulating their cognitive and emotional resources, resulting in lower mental fatigue and potentially leading to improved academic performance. Therefore, it is reasonable to infer that individuals with better health status generally experience fewer challenges and have more mental resources and energy to address the cognitive demands of coursework (Mosleh et al., 2022).

The results also suggest that chronic conditions have a direct, negative impact on expected grades, but not on mental fatigue. Students with chronic conditions usually reported poorer health and lower grade expectations, highlighting the difficulties they experience balancing health and academic pursuits (Kirkpatrick, 2020). Compared to their healthy peers, these students face academic challenges that can lead to disengagement and lower attainment over time, increasing the likelihood of failure and lowering grade expectations (Lum et al., 2019). Moreover, lower educational expectations from teachers and parents can have a negative impact on the long-term self-efficacy and self-confidence of students with chronic conditions (Cerqueira et al., 2022). The absence of a significant direct relationship between chronic conditions and mental fatigue suggests a more complex, indirect association, potentially mediated by factors like perceived health status. While chronic conditions may not directly cause mental fatigue, it can contribute to perceived health status and general sense of fatigue, which in turn may increase mental fatigue. This aligns with the motivational control theory of fatigue, proposing that individuals with chronic conditions may adjust their efforts and expectations to conserve physical and mental well-being, potentially masking the direct impact of chronic conditions on mental fatigue (Hockey, 2013).

While the path model is a useful tool for understanding the complex relationships between factors that may impact student learning outcomes, it is important to remember that it is not a definitive causal relationship. When interpreting the results, it must be considered that the relationships shown in the model are associations and possible influences and not experimental causal claims. It is also important to consider that the relationships may be more complicated than represented in the model and that there may be reciprocal relationships between some of the factors. Another area to consider is that while the model presents results on how chronic conditions and health status can negatively impact academic success, it is important to recognize that this is not the case for all students, and that does not mean that students with these conditions will not succeed. Many students with health issues overcome academic challenges effectively and achieve success.

### 4.1. Implications

The results suggest that supporting students’ physical and mental health is critical not only to their well-being but also to their academic success. Therefore, it is crucial that the institute develops and offers mentally friendly online courses to accommodate student individual differences and foster an inclusive learning environment (Inan et al., 2024). Research has shown that a variety of interventions focused on course design and enhancing students’ activities have been developed and studied to improve student mental health and reduce mental fatigue. For example, a study conducted in Germany by J. M. Basch et al. (2025) showed that promoting active student participation, such as student discussions, providing polls, and nonverbal feedback during online courses, significantly reduced fatigue. A viable strategy for mitigating mental fatigue also involves adjusting academic demands to match students’ mental resources. Therefore, course content, activities, and assessments should be designed to minimize cognitive overload, promote sustained attention through the inclusion of breaks, the provision of flexible deadlines, and the use of different teaching methods to create manageable learning experiences for students in online learning environments (McGowan, 2022).

Promoting health practices, specifically physical activity, is another key area. Active engagement in physical education improves both cognitive and non-cognitive aspects of students’ health in addition to their physical and mental well-being (Biddle et al., 2019). Numerous research studies have found that physical activity and exercise programs can effectively alleviate students’ fatigue (Erlacher et al., 2015; Teuber et al., 2024). Other effective ways to reduce online student fatigue include individual and group-centered mental well-being interventions such as awareness-enhancing strategies, typically self-monitoring of stress symptoms and psychoeducation (Le Blanc & Schaufeli, 2008). Moreover, practices like mindfulness-based interventions can reduce student fatigue (Wang et al., 2024).

The model highlights the complex interplay of factors influencing student academic expectations and performance, emphasizing the importance of addressing mental fatigue, health, and chronic conditions in online learning environments. Therefore, institutions should adopt a comprehensive approach to student well-being, addressing both physical and mental health needs. This includes creating a supportive and inclusive environment where students feel comfortable seeking help, implementing programs and policies promoting mental health, stress reduction, and healthy habits, and offering flexible learning options and accommodations. The recent rapid expansion of mental health services has been encouraging, but the integration of online students into these support systems remains a challenge (Garrett et al., 2023). This is likely due to several factors, including the fact that online students may be less informed about mental health services and, most likely, that mental health service providers are less familiar with the needs and unique challenges faced by online students.

The model’s framework can guide future research to explore the complex relationships between student mental fatigue, learning outcomes, and other relevant variables such as commitments, coping mechanisms, social support, and access to resources. By examining these relationships in more detail and employing rigorous causal testing methods, researchers can gain a more comprehensive understanding of these factors and develop effective interventions to support students’ well-being and academic success. Future studies should consider the multifaceted nature of student health and fatigue, which encompass physical, mental, and emotional sub-dimensions, each potentially influencing learning outcomes. Longitudinal studies are needed to examine changes in mental fatigue, health, and academic expectations over time. Additionally, future research could explore capturing student mental fatigue through objective measures like electroencephalography and heart rate variability, in addition to self-reported data (Bayne & Inan, 2022). Finally, to enhance the generalizability of the findings, future studies could include students from diverse settings and educational levels, such as K-12 students, graduate students, and other learner groups.

### 4.2. Limitations

Several limitations of this study should be considered when interpreting the results. Firstly, the sample exhibited a significant gender imbalance, with a substantially larger proportion of female participants compared to male participants. Several factors may have contributed to this disparity. Firstly, there is a documented trend of higher female enrollment in higher education institutions (National Center for Education Statistics, 2023), and this trend is even more pronounced in online learning environments. According to a recent report, women constitute a significant majority of both undergraduate and graduate online college student populations, accounting for nearly two-thirds of enrolled students (Aslanian & Fischer, 2023). Secondly, research suggests that women are more likely to participate in surveys than men (Becker, 2022). This difference in survey response rates could further contribute to the higher representation of female participants in our study. This disproportionate gender representation limits the generalizability of our findings and poses a significant challenge to conducting meaningful subgroup analyses (Mazza et al., 2025). This gender imbalance should be considered when interpreting our findings, and future research with more balanced samples is necessary to investigate potential gender-specific effects.

This study relied on self-reported data for other constructs as well, such as mental fatigue and health status, which is subject to recall bias and social desirability bias (Althubaiti, 2016). Participants’ assessments may not perfectly reflect their actual experiences. Future research could incorporate objective measures of these constructs, such as physiological indicators or medical records where possible, to complement self-report measures. In addition, this study employed a single item to evaluate health status, which asked students about their overall perceived health. While this approach offers a general understanding of students’ health perspectives, it is crucial to acknowledge the absence of specifically validated instruments for assessing such broad perceptions of health, especially within the context of online learning. Ideally, comprehensive medical data would offer a more precise measure of students’ health status. However, collecting such detailed medical information is not feasible nor practical for large-scale survey-based studies due to privacy concerns, logistical challenges, and the voluntary nature of participation.

While path analysis allows us to evaluate the hypothesized model and relationships, it does not establish causation. The observed associations between variables may be bidirectional or influenced by unmeasured confounding factors (Kline, 2016). Longitudinal studies are needed to examine the temporal ordering of these relationships and to establish causal pathways. Furthermore, our sample was primarily consisted of undergraduate students in the United States. This may limit the generalizability of our findings to other populations, such as graduate students or students in other countries. Future research should aim to include more diverse samples to enhance the external validity of the findings.

## 5. Conclusions

Overall, this research provides a more nuanced understanding of the complex interplay between health, mental fatigue, and academic expectations. The results show that students with higher levels of mental fatigue and chronic conditions tend to report lower expected grades, and that poor physical health indirectly affects grade expectations by contributing to increased mental fatigue. This study makes several important contributions to the research community. First, it confirms the association between poor health and increased mental fatigue, supporting previous research indicating that poor health negatively impacts cognitive ability and academic performance. Second, it highlights the mediating role of mental fatigue between health status and academic expectations, suggesting that addressing mental fatigue is critical to improving student outcomes, particularly for those with health challenges. Third, by uncovering specific relationships between factors affecting student success, this research underscores the importance of holistic support and paves the way for targeted interventions to promote both physical and mental health, leading to improved academic outcomes.

## Figures and Tables

**Table 1:** Correlations, Means, and Standard Deviations (N = 418).

Variables	M	SD	Expected Grade	Mental Fatigue	Health Status	Chronic Conditions
1. Expected Grade	4.21	0.76	1			
2. Mental Fatigue	3.21	0.92	−0.29	1		
3. Health Status	3.25	0.92	0.17	−0.22	1	
4. Chronic Conditions	0.18	0.38	−0.14	−0.04	−0.23	1

**Table 2:** Direct and Indirect Effects of the Factors (Standardized Coefficients).

	Endogenous (Dependent) Variables								
Variables	Health Status			Mental Fatigue			Expected Grade		
	Direct	Indirect	Total	Direct	Indirect	Total	Direct	Indirect	Total
1. Chronic Conditions	−0.23 *	--	-	−0.01 ^ns^	0.05 *	0.04 ^ns^	−0.11 ^	−0.03 ^ns^	−0.14 *
2. Health Status	--	--	--	−0.23 *	--	--	0.09 ^ns^	0.06 *	0.15 *
3. Mental Fatigue	--	--	--	--	--	--	−0.27 *	--	--
*R* ^2^			0.05			0.05			0.11

Note: * *p* < 0.01, ^ *p* < 0.05, ^ns^ = Not Significant.

## Data Availability

The data that support the findings of this study are available from the corresponding author upon reasonable request.

## Abbreviations

The following abbreviations are used in this manuscript:

CFI	Comparative Fit Index
RMSEA	Root Mean Square Error of Approximation
TLI	Tucker–Lewis Index
SMFS	Student Mental Fatigue Survey
SRMR	Standardized Root Mean Square Residual
